# Circulating Long and Circular Noncoding RNA as Non-Invasive Diagnostic Tools of Hepatocellular Carcinoma

**DOI:** 10.3390/biomedicines9010090

**Published:** 2021-01-19

**Authors:** Caecilia H. C. Sukowati, Loraine Kay D. Cabral, Claudio Tiribelli, Devis Pascut

**Affiliations:** 1 Fondazione Italiana Fegato ONLUS, AREA Science Park, Campus Basovizza, SS14, km 163.5, 34149 Trieste, Italy; caecilia.sukowati@fegato.it (C.H.C.S.); kay.cabral@fegato.it (L.K.D.C.); ctliver@fegato.it (C.T.); 2 Doctoral School in Molecular Biomedicine, University of Trieste, 34100 Trieste, Italy

**Keywords:** hepatocellular carcinoma, ncRNAs, lncRNA, circRNA, non-invasive biomarker, diagnosis

## Abstract

Hepatocellular carcinoma (HCC) is one of the most common causes of cancer-related death worldwide, partially due to late diagnosis of the disease. Growing evidence in the field of biomarker discovery has shown the promising use of nucleic acid in the early detection of many cancers, including HCC. Here, we review data on how various long noncoding RNAs (lncRNAs) and circular RNAs (circRNAs) could be used as a diagnostic tool for HCC being differentially expressed in HCC compared to non-HCC patients. These non-coding RNAs (ncRNAs) showed high stability in the blood being present as free-circulating molecules or encapsulated into exosomes. This review reports some recent evidence on the use of lncRNAs and circRNAs as possible diagnostic biomarkers for HCC. Further, their pathophysiological mechanism in liver carcinogenesis was also described, elucidating the complex regulatory networks making these ncRNAs of particular relevance for the study of liver malignancy cancer.

## 1. Hepatocellular Carcinoma

Hepatocellular carcinoma (HCC) is one of the most common cancers worldwide, ranking as the sixth most common malignancy and the fourth most common cancer-related death, for both sexes [1,2]. The incidence and the mortality rates are comparable (840,000 and 780,000 per year, respectively, GLOBOCAN 2018), pointing to a high mortality risk for the disease.

HCC is a heterogeneous malignancy, often occurring in a setting of cirrhosis caused by multiple etiological factors. Major risk factors for HCC are chronic infection of hepatitis B virus (HBV) and/or hepatitis C virus (HCV), alcoholic liver disease, non-alcoholic fatty liver disease/non-alcoholic steatohepatitis (NAFLD/NASH), and exposure to aflatoxin. Even though active hepatitis C and B chronic infection continue to drive most of the global burden of HCC, the booming rising of the metabolic syndrome manifested to NAFLD/NASH is a major cause of liver disease that may lead to the development of HCC [3,4]. Overall, one-third of cirrhotic patients will develop HCC [5].

The management of HCC is based on a standard protocol on the surveillance, diagnosis, and treatment recommendation of international guidelines [6,7,8]. The European guideline (Barcelona Clinic Liver Cancer/BCLC) strongly recommends non-invasive criteria for HCC diagnosis in a cirrhotic setting, while the diagnosis must be confirmed by pathology in non-cirrhotic livers [6]. For the very early stage HCC (BCLC 0) and early-stage (BCLC A), curative treatment such as liver resection, liver transplantation, and ablation [4] are recommended since they result ina five-year survival higher than 50% [9]. For the intermediate stage (BCLC B), transarterial chemoembolization (TACE) was considered the first-line treatment, with a median survival of around 40 months in well-selected candidates, with a state-of-the-art technique and a super-selective approach based on the strict evaluation of tumor characteristics including tumor number, size, and liver function. Patients in an advanced stage can receive systemic therapies, Sorafenib as a first-line drug [10], while patients in the terminal stage can only receive best supportive care with median survival of three to four months or 11% at one year [11]. Early diagnosis of HCC is fundamental for the improved survival of the patients, summarized in [6].

In cancer research, including in HCC, noninvasive liquid biopsy has become an attractive modality for the early detection of cancer. Liquid biopsy is a method where biomarkers in bodily fluid samples are used to determine the pathophysiological state of an individual. It is commonly used for the diagnosis, prognosis, monitoring, and treatment choice of a disease.

Serum alpha-fetoprotein (AFP) is still the most common serum biomarker for HCC screening and diagnosis. However, the use of AFP alone is limited since not all HCC cells that secrete AFP and AFP may be elevated in cirrhosis or hepatitis [12]. A multicenter study with more than 1100 HCC subjects showed a low sensitivity (54%) of AFP in the diagnosis of HCC and a prognostic value area under the curve (AUC) of 0.59 [13]. A previous systematic review reported that, using a cut-off of 20 ng/mL, AFP showed a specificity of 80–92%, but a poor sensitivity of 41–65% [14].

Cancer by-products, including circulating tumor cells (CTCs), cell-free nucleic acids (cfDNA and cfRNA), extracellular vesicles (EVs), and tumor-derived metabolites, are common biomarkers used to detect the presence of malignancy. Both cfDNA and cfRNA are released into the circulation via various systems, including active secretion or means of the apoptosis, necrosis, and lysis of CTCs [15]. The presence of these molecules in the fluid (usually in peripheral blood) can be used to represent the cancer tissue of origin. Together with its non-invasive approach, cfRNA biomarkers are more dynamically regulated, tissue-specific, and abundant in extracellular environments [16].

Many studies reported the significance and diagnostic power of microRNAs (miRNAs) biomarkers for HCC diagnosis. However, little is known about the presence of long cfRNA in circulation. This review presents available information on long cfRNAs, in particular the long non-coding RNA (lncRNA) and circular-RNA (circRNA) for early diagnosis of HCC.

## 2. Non-Coding RNAs

In the beginning, cancer studies were mainly based on the central dogma of biology focusing on protein-coding genes. In the last decades; however, discoveries of novel structural and functional nucleic acids open up new knowledge in biology and medicine. Advances in high-throughput nucleic acid technologies facilitates a systematic screening and possible discovery of various new molecules. The human genome is pervasively transcribed and produces thousands of regulatory non-protein-coding RNAs (ncRNAs), including microRNAs (miRNAs), small interfering RNAs (siRNAs), PIWI-interacting RNAs (piRNAs), circular RNA (circRNA), and various classes of long ncRNAs [17]. These ncRNAs were plastic and they are capable of complex mechanisms in a broad- and sequence-specific manner [17].

### 2.1. Free-Circulating lncRNAs

The study from Iyer et al. (2015) showed that, from a consensus of around 91,000 human genes, over 68% of genes were classified as long noncoding RNAs (lncRNAs), of which 79% were previously unannotated [18]. The lncRNAs, non-protein-coding transcripts with length > 200 nucleotides (nt), is the broadest class in non-coding RNA, including mRNA-like ncRNAs [17]. It is involved in multiple mechanisms in an organism. Accumulating evidence showed that these functional RNA molecules were important regulatory molecules in many, if not all, biological processes across organisms [19]. Multiple functions of this RNA included the recruitment of chromatin-modifying enzymes or interaction with proteins to direct their binding to DNA, also in epigenetic regulation of protein-coding gene expression [19,20].

LncRNAs contribute to the pathogenesis of numerous human diseases. In the liver, the first evidence of lncRNA, the H19, was found in fetal liver tissue. Cellular fractionation showed that H19 RNA is cytoplasmic but not associated with the translational machinery, thus it was not a classical mRNA [21]. Further, various lncRNAs were differentially expressed in cancers including in HCC [22,23,24]; however, their biological functions are complex and are not fully elucidated.

In HCC, lncRNAs could act as a modulator of the liver microenvironment and chronic liver diseases including viral infection, DNA and protein binding transcriptional activation, and miRNA and mRNA sponging, and encoding small peptides (comprehensively reviewed in [22]). Recent evidence showed also that the dysregulation of lncRNAs, such as lncMAPK6, LncSox4, and Linc00210, promoted the genesis of tumor-initiating cells (TICs), leading to HCC development [25,26,27]. Linc00210 drove the self-renewal and propagation of liver TICs through activating Wnt/β-catenin signaling, one of the most common dysregulating oncogenic pathways in HCC [26].

LncRNAs can be detected in the serum and plasma of an individual. Further, lncRNAs were reasonably stable for molecular analysis upon blood withdrawal. lncRNAs were not significantly degraded when samples were subjected to physical (prolonged room temperature incubation and multiple freeze-thaw cycles), chemical (low/high pH), and biological (RNase A) stress [28,29]. In pathological conditions, their presence and expression were altered thus can be used as a biomarker for diagnosis, disease monitoring, development of molecular-targeted therapy, and study of genetic heterogeneity [15]. A growing body of evidence showed that ncRNAs, including lncRNAs, may be primary genetic regulators in complex animals making them ideal diagnostic markers [17]. Here we reported lncRNAs for HCC diagnosis described in two or more studies by different research groups, including the HULC, LINC00152, UCA1, and MALAT1. Various lncRNAs useful in HCC diagnostics based on the calculation of the receiver operator characteristic (ROC) are shown in Table 1.

HULC (highly upregulated in liver cancer) is one of the first and most studied lncRNAs in HCC. Discovered in 2007, HULC was sequenced upon screening of the HCC-specific gene library of deregulated genes in HCCs, focal nodular hyperplasias, and cirrhosis utilizing cDNA arrays. HULC was also found to be present in peripheral blood cells [30]. Further studies showed multiple properties of HULC in promoting the growth and metastasis of HCC cells [31,32] and autophagy attenuated the sensitivity of HCC cells to chemotherapeutic agents via HULC/USP22/Sirt1 mechanism [33]. Several meta-analysis studies demonstrated that HULC can be used in the prognosis of various types of cancer. A higher HULC expression was associated with overall survival, lymph node metastasis, distant metastasis, and tumor stage [34,35,36,37,38].

Further research showed that circulating HULC in HCC patients could be used as a diagnostic marker, being highly up-regulated in patients with HCC compared to healthy individuals [39,40,41], with the area under the curve (AUC) of the receiver operator characteristic (ROC) curve around 0.80. Several studies; however, did not find up-regulation of HULC in HCC samples set [16,42]. A recent review summarized the role of HULC in carcinogenesis [43].

**Table 1:** List of long non-coding RNAs (lncRNAs) and circular RNAs (circRNAs) for hepatocellular carcinoma (HCC) diagnosis through a receiver operator characteristic (ROC) curve analysis.

	n Samples ^a^	Type	Exp	AUC ^b^	SEN/SPE (%) ^b^	AUC ^c^	SEN/SPE (%) ^c^	Ref
lncRNA								
HULC	30/-/20	plasma	up	0.860	na	-	-	[40]
HULC	129/76/93	serum	up	0.796	86/62	0.756	86/56	[41]
HULC	66/-/52	plasma	up	0.780	na	-	-	[39]
LINC00152	129/76/93	serum	up	0.895	78 / 89	0.877	81/83	[41]
LINC00152	100/100/100	plasma	up	0.869	na	0.826	na	[42]
LINC00152	66/-/52	plasma	up	0.850	na	-	-	[39]
MALAT1	129/76/93	serum	up	0.768	60/81	0.733	60/76	[41]
MALAT1	88/28/51	plasma	up	na	na	0.666	51/89	[44]
RP11–160H22.5	217/100/250	plasma	up	0.601	na	na	na	[45]
RP11–160H22.5	100/100/100	plasma	up	0.884	na	0.859	na	[42]
XLOC014172	217/100/250	plasma	up	0.866	na	na	na	[45]
XLOC014172	100/100/100	plasma	up	0.759	na	0.735	na	[42]
LINC00974	150/-/na	plasma	up	0.733	-	-	51/96	[46]
UCA1	129/76/93	serum	up	0.858	81/75	0.809	67/81	[41]
UCA1	70/32/38	serum	up					[47]
UCA1	105/105/105	serum	up	0.902	73/99	0.848	71/94	[48]
UCA1	82/34/44	serum	up	0.861	93/82	0.728	61/74	[49]
XIST	74/60/72 (female)	mononuclear cells	up	0.815	84/73	0.802	73/82	[50]
XIST	74/60/72 (female)	granulocytes	up	0.829	73/84	0.782	72/83	[51]
TSIX	65/34/32	serum	up	0.866	88/73	na	na	[52]
PTTG3P	129/76/93	serum	up	0.785	83/61	0.768	83/57	[41]
SPRY4-IT1	129/76/93	serum	up	0.808	77/71	0.768	77/68	[41]
SPRY4-IT1	60/85/63	plasma	up	0.702	87/50	0.611	44/87	[28]
UBE2CP3	129/76/93	serum	up	0.812	88/62	0.756	78/60	[41]
LINC00161	24/32/56	serum	up	0.794	75/73	na	na	[53]
LOC149086	217/100/250	plasma	up	0.759	na	na	na	[45]
RN7SL1 S fragment	100/-/50	plasma	up	0.869	90/70	-	-	[16]
SNHG1	72/50/50	plasma	up	0.920	65/95	0.740	65/95	[54]
uc003wbd	137/104/138	serum	up	0.860	na	0.700	na	[55]
AF085935	137/104/138	serum	up	0.960	na	0.860	na	[55]
CTBP	78/36/44	serum	up	0.910	91/89	0.830	91/89	[56]
uc001ncr	50/50/50	serum	up	0.886	88/79	0.886	88/79	[57]
AX800134	50/50/50	serum	up	0.925	93/87	0.925	93/87	[57]
WRAP53	82/34/44	serum	up	0.896	85/82	0.787	85/71	[57]
PTENP1	129/76/93	serum	down	0.602	89/71	0.530	89/23	[41]
JPX	42/-/68	plasma	down	0.814	52/100	-	-	[50]
circRNA								
hsa_circ_0000798	30/-/72	pbmc	up	0.70	na			[58]
hsa_circ_104075	60/-/101			0.97	96/98			[59]
hsa_circ_0008234; circ-FOXP1	16/-/30	serum	up	0.93	na			[60]
CircSMARCA5	103/262/135	plasma	down	0.94	87/89	0.85 ch 0.71 cirr	75/89 ch 77/64 cirr	[61]
hsa_circ_0003998	50/50/100	plasma	up	0.89	80/84	0.83	83/70	[62]
hsa_circ_0027089	72/40/64	plasma	up	0.79	58/85	0.77	57/85	[63]
hsa_circ_0051443	60/-/60	exosome	down	0.81	na			[64]
hsa_circ_0004001	32/-/21	serum	up	0.73 *	76/81			[65]
hsa_circ_0004123	32/-/21	serum	up	0.79 *	67/84			[65]
hsa_circ_0075792	32/-/21	serum	up	0.76 *	81/69			[65]
hsa_circ_0009582	200/-/200	plasma	up	0.80	na			[66]
hsa_circ_0037120	200/-/200	plasma	up	0.84	na			[66]
hsa_circ_0140117	200/-/200	plasma	up	0.85	na			[66]

^a^ Sample (HCC/chronic&cirrhosis/healthy) circulation-derived samples only; ^b^ vs healthy control; ^c^ vs chronic/cirrhosis; na = not available; AUC = area under the curve; * Calculated based on exoRBase data (http://www.exorbase.org/exoRBase/toIndex).

Another well studied lncRNA, the LINC00152 (long intergenic non-protein-coding RNA 52), was found through gene trapping technique, showing its role in the invasion and migration of HCC cells [67]. LINC00152 was involved in hepatocarcinogenesis by the activation of mTOR pathway [68]. It was hypomethylated in tumor tissues and human HCC cell lines [68] and during hepatocarcinogenesis [69]. In the blood, the expression level of LINC00152 increased from normal healthy control to chronic hepatitis B, liver cirrhosis, being highest in HCC [41,42]. Among other lncRNAs including HULC, LINC00152 was found to be the best diagnostic lncRNA with high AUC and sensitivity/specificity values [41]. Plasma and exosomal LINC00152 were also significantly elevated in gastric cancer patients compared with healthy controls, indicating its use in the cancer diagnosis [70].

Urothelial cancer associated 1(UCA1) was first identified in bladder cancer [71]. UCA1 had been abundantly investigated as a non-invasive biomarker for various types of cancer, in particular for bladder cancer [72,73,74,75], both in blood and urine. The presence of UCA1 in body fluid was associated with tumor characteristics and patients’ prognosis. In HCC, it was up-regulated in HCC tissues and was associated with several clinical features and malignant behaviors in HCC [76,77]. A meta-analysis of seven studies validated the result, showing the association of UCA1 expression with tumor size, metastasis, and overall survival [78]. Several studies demonstrated the high expression of UCA1 in sera samples of HCC patients [41,47,48,49]. These studies showed a good AUC value > 0.800.

Metastasis-associated lung adenocarcinoma transcript 1 (MALAT1) showed significant upregulation in neoplastic samples (HCC and highest in hepatoblastoma) compared to adjacent tumor and normal tissues [79]. Patients with high MALAT1 expression had a significantly increased risk of tumor recurrence after liver transplantation, pointing MALAT1 as an independent prognostic factor. MALAT1 inhibition in HepG2 cells reduced cell viability, motility, invasiveness, and increased the sensitivity to apoptosis [80]. Another analysis on HepG2 cells showed that MALAT1 involved in the HCC progression by sponging and competitive binding to miRNA-204, and released the suppression on sirtuin 1 (SIRT1) [81]. MALAT1 expression has been used in cancer diagnosis, including in bladder and lung cancer [82,83,84]. On the contrary, there is still limited information in HCC. Plasma MALAT1 was up-regulated in HCC compared to hepatic diseases (chronic viral hepatitis and NAFLD/NASH), and it can be used to distinguish HCC from other liver diseases [44]. A recent 2020 study showed that serum MALAT1 expression could distinguish HCC from healthy individuals and chronic hepatitis with AUC 0.768 and 0.733, respectively. However, these values were lower compared to HULC and LINC00152 [41].

RP11–160H22.5 was a novel lncRNA firstly detected in HCC upon global screening of lncRNA in plasma of HCC patients and the cancer-free individuals using a microarray platform. Microarray data was then confirmed by RT-PCR for 20 and 147 plasma HCC cases in training and validation sets, respectively. In the validation test; however, the AUC for this lncRNA was significantly decreased (0.900 to 0.601) in distinguishing HCC from the healthy control [45]. Another study comprised of 100 HCC cases supported the result with an increased AUC value to 0.884, indicating this lncRNA as a diagnostic biomarker for HCC [42]. Until now, there is no available data on its functional role in the HCC progression.

SPRY4 intronic transcript 1(SPRY4-IT1) was up-regulated in HCC tissues and its high expression was associated with poor prognosis of the patients [85]. SPRY4-IT1 promoted the epithelial-mesenchymal transition (EMT) by up-regulating the transcription factor and EMT markers via interaction with EZH2 [86]. Recently, the expression of SPRY-IT1 was found to have diagnostic value to distinguish HCC patients from healthy controls and chronic hepatitis patients with AUC of 0.81 and 0.78, respectively [41]. Previously, SPRY4-IT1 was included in a lncRNAs panel together with MALAT1 and PCAT-1 for the non-invasive diagnosis and recurrence prediction of bladder cancer [87].

In several other studies, various lncRNAs, such as XLOC04172, LINC00974, TSIX, UBE2CP3, SNHG1, uc003wbd, AF085935, CTBP, uc001ncr, AX800134, WRAP53, and JPX, were also able to distinguish HCC samples from healthy donors and patients with chronic hepatitis or cirrhosis with acceptable and even good AUC values of above 0.800 (Table 1).

### 2.2. Free-Circulating CircRNAs

Circular RNAs (circRNAs) were recently identified as an endogenous class of non-coding RNAs (ncRNAs) having multiples functions in the regulation of gene expression [88,89,90]. They were originated from a pre-mRNA undergoing the so-called “head-to-tail” or lariat-driven circulation, or back splicing process in which the splice donor site of a downstream exon of a protein-coding mRNA binds to the splice acceptor site of an upstream exon [91,92] to produce a circular product.

Most circRNAs derive from known protein-coding genes and consist of a single exon, multiple exons, exon, and intron (in this case the circular RNA is named EIciRNAs) or only introns (named ciRNAS) [93]. Generally, circRNAs localize in the cytoplasm [94], while ciRNAs and EIciRNAs reside in the nucleus where they can interact with U1 snRNP and RNA polymerase II to promote transcription of their parental genes. CircRNAs can compete also for the binding to the splicing machinery of their parental genes, thus inhibiting the full maturation [95]. Besides the roles in the nucleus, cytosolic circRNAs can bind and sequester specific proteins to appropriate subcellular positions, and participate in the modulation of some protein-protein or protein-RNA interactions [96]. Lastly, circRNAs are also known to work as competitive endogenous RNAs (ceRNAs) sponging intracellular microRNAs (miRNAs) [97,98]. As per lncRNA, circRNA can be detected in the serum and plasma of an individual. The use of circRNAs as a diagnostic tool for HCC is still recent, thus this review describes proposed circRNAs in single studies in current literature (Table 2). In the future, independent comparison to other studies will be needed.

Besides its role in activating yes1 associated transcriptional regulator (YAP1) as a ceRNA to bind the YAP inhibitor miR-582-3p, the hsa_circ_104075 was found to be up-regulated in serum of HCC patients, compared to healthy controls. It showed better performances as a predictor for HCC (AUC 0.973) compared to other non-coding RNAs, including miRNAs, and classical protein biomarkers such as AFP (AUC 0.750) [59]. Likewise, the circRNA generated from the Forkhead Box P1 gene (FOXP1), circ-FOXP1, was highly expressed in the serum of HCC patients, being consistent with its expression in tumoral tissue [60]. Considering its molecular role as ceRNA for two oncosuppressor miRNAs, miR-875-3p and miR-421, the high levels of circ-FOXP1 were associated with larger tumors, microvascular invasion, and lower survival. Despite being analyzed in a low number of samples, it was able to distinguish cancer patients from controls with an AUC of 0.930, suggesting some potential value as a diagnostic biomarker in HCC [60].

In 2018, Gong et al. [102] identified circ-ZEB1.33 being significantly higher in serum of HCC patients compared to healthy controls, and its levels were positively correlated with the increase of TMN stage, being higher in the III–IV stage compared to the I–II stage. Based on this evidence, the authors suggested serum circ-ZEB1.33 as a diagnostic biomarker for HCC, as it was up-regulated also in tumor tissues. In HCC, circ-ZEB1.33 sponged miR-200a-3p ensuring the expression of the miR-200a-3p target, cyclin dependent kinase 6 (CDK6) [102], that had an active role in phosphorylating the tumor suppressor protein, retinoblastoma transcriptional corepressor 1 (Rb1), thus promoting the cell cycle enter into S phase.

The investigation of the circSMARCA5 expression in HCC plasma samples, as well as in precancerous conditions, such as cirrhosis and hepatitis B and C, evidenced the progressive down-regulation of this circRNA from hepatitis to cirrhosis and HCC [61]. ROC curve analysis evidenced the potential of this circRNA in distinguishing patients with hepatitis or cirrhosis from patients with HCC, especially those patients with AFP levels below 200 ng/mL. Besides, the comparison with healthy subjects further proved the value of circSMARCA5 in distinguishing HCC patients from controls. Tissue analysis proved the tumor origin of circSMARCA5, in which it is supposed to promote metalloproteases expression (MMP7 and MMP9), as evidenced from in vitro experiments conducted in Huh7 cells. Indeed, the decrease of circSMARCA5 in tumors was correlated with invasion, tumor differentiation, and TNM stage [61].

On the contrary, the plasma levels of hsa_circ_0003998 in HCC patients were significantly higher compared to the levels in hepatitis B patients and healthy controls, being able to significantly distinguish HCC patients from the other two groups with an AUC greater than 0.80. Importantly, the plasma levels of hsa_circ_0003998 were significantly correlated with higher serum AFP level, larger tumor diameter, microvascular invasion (MVI), and lower differentiation level [62].

Three circRNAs, hsa_circ_0004001, hsa_circ_0004123, and hsa_circ_0075792, investigated in the serum of 71 HCC patients, showed marked upregulations compared to 40 healthy individuals [86]. Despite this study being performed in whole serum RNA extracts, consistency was found with the data retrieved from the exoRBase database [109] (http://www.exorbase.org/exoRBase/toIndex), which include exosomal circRNAs from 21 HCC patients. Indeed, the diagnostic potential of these three candidates was also verified through ROC curve analysis, showing the discriminatory potential of the circRNAs between HCC patients and controls with an AUC always above 0.70 [65].

Three recent and comprehensive studies investigated the circRNAs profile in plasma samples of HBV-related HCC and controls. In one of these studies, 13,617 circRNAs were profiled through microarray in 10 HBV-related HCC patients, compared to five patients with HBV-related cirrhosis [63]. They identified 157 up-regulated and 161 downregulated circRNAs in HCC compared to cirrhosis, where 86 were able to cluster HCC samples vs. cirrhosis samples. In the training phase, six circRNAs were confirmed to be differentially expressed between HCC and cirrhosis, with hsa_circ_0077930, hsa_circ_0027089, hsa_circ_0001818, and hsa_circ_0026337 being up-regulated, while hsa_circ_0011883 and hsa_circ_0001070 were downregulated. In particular, one of them, hsa_circ_0027089 was further validated in a cohort of 64 HCC patients, 40 cirrhosis patients, and 72 healthy individuals, resulting in significant up-regulation in the plasma of patients with HCC compared to the other groups [63]. Besides, its diagnostic value in distinguishing HCC from cirrhosis was supported by a ROC curve analysis that found an AUC of 0.77 and 0.79, when comparing HCC patients vs. patients with cirrhosis and healthy controls, respectively [63].

Similarly, Wu et al. [66], through a microarray approach, identified five circRNAs that were significantly up-regulated in HCC patients compared to patients with CHB. They were decreased after surgical removal of the nodules, thus underlying a possible direct link with the presence of a tumor. Among the five candidates, hsa_circ_0009582, hsa_circ_0037120, and hsa_circ_0140117 resulted significantly up-regulated in the validation group, consisting of 200 HCC patients compared to 200 patients with CHB, and 200 healthy controls, and the increase extent of positively was correlated with the progression of the disease [66]. Importantly, the combination of these three candidates was able to significantly distinguish HCC patients from healthy controls with a positive predictive value (PPV) and negative predictive value (NPV) of 80% and 95%, respectively, and from CHB patients with a PPV and NPV of 84% and 80%, respectively [66]. Various circRNAs useful in HCC diagnostics based on the calculation of the ROC curve are shown in Table 1.

A different panel of plasma circRNA, named CircPanel and consisting of hsa_circ_0000976, hsa_circ_0007750, and hsa_circ_0139897, was suggested by Yu and colleagues for the diagnosis of HBV-related HCC (*n* = 600), even at early stages with solitary small nodules less than 3 cm [108]. In addition, the CircPanel showed higher accuracy than AFP in distinguishing individuals with HCC from non-HCC subjects, as well as seen for lncRNAs.

In many studies, the use of lncRNA expression level, especially the LINC00152, was found to have a better diagnostic power compared to that of AFP alone. However, majority of the cited studies, if not all, showed that the combination between various ncRNAs and serum AFP level increased the diagnostic value (Table 3).

## 3. Exosomal-Derived lncRNAs and CircRNAs

**Figure 1:**
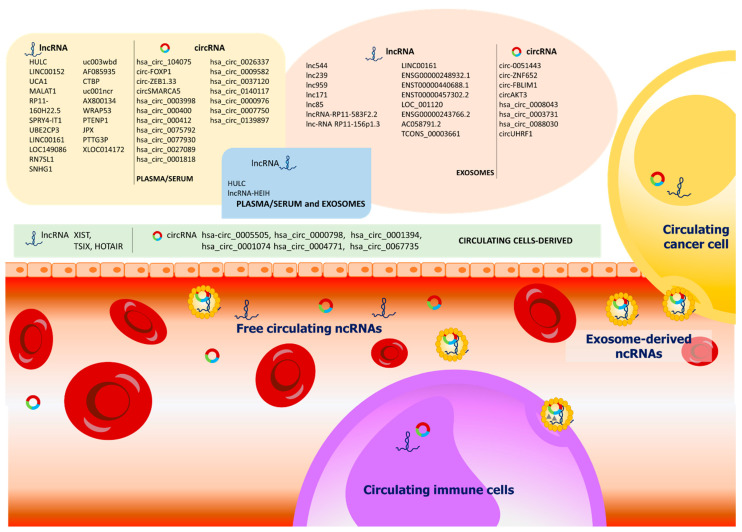
Circulating long non-coding RNAs (lncRNAs) and circular RNAs (circRNAs) for hepatocellular carcinoma (HCC) diagnosis. Both lncRNAs and circRNAs can be found as free-circulating molecules or encapsulated into exosomes. Circulating cancer cells and immune cells can release exosomes contributing to the variety of circulating non-coding RNAs (ncRNAs) present in the bloodstream.

Besides the presence of cfRNA in plasma or serum, various studies indicated that cfRNAs exist in extracellular vesicles (EVs), such as exosomes and microvesicles (Figure 1). EVs act as a carrier ofa heterogeneous cargo of proteins, signaling lipids, and nucleic acids from donor cells to recipient cells, and thus had been proposed to serve as mediators of cell-to-cell communication.

Exosomes are frequently associated with cancer. They are the smallest subtype of EVs with 30 to around 100 nm in diameter, surrounded by a phospholipid bilayer and as spherical- to cup-shaped nanoparticles. They have specific surface molecular characteristics CD9, CD81, and CD63, LAMP1, and TSG101 [111,112]. Exosomes are formed by the inward budding of multivesicular bodies and are released into the microenvironment, interstitial spaces, and bodily fluids following the fusion with the plasma membrane [112]. Studies showed the presence of RNAs in EVs, both coding and non-coding RNAs (reviewed in [113]). The detection of exosomal-ncRNA is a good approach, since RNAs inside the exosome are protected from RNases, thus it can be presumed that the integrity and function of the RNAs are not altered [70,114].

Since ncRNAs are enriched and stable in exosomes they were considered a class of circulating RNA to be included in the biomarker discovery studies. The first evidence in this regard derives from the study of Li and colleagues [115] that identified a profile of exo-circRNAs able to distinguish colon cancer patients from healthy controls. Further evidence then identified circRNAs in HCC-associated exosomes, opening new perspectives for the identification of circulating RNAs as biomarkers for this disease.

Cytosolic lncRNA and circRNA can be sorted into the exosomal cargo to be released together with the multitude of other non-coding regulatory RNAs, mRNAs, and proteins into the bloodstream. Thousands of different ncRNAs are known to be included in exosomes derived from several cancer types, including HCC [109].

### 3.1. Exosome-Derived lncRNAs

A recent study by Huang using RNA sequencing identified around 8500 lncRNAs that were differentially expressed (DE-) in plasma exosomes between HCC patients and healthy controls [116]. Here, the authors analyzed the best five candidates of novel DE-lncRNAs (lnc544, lnc239, lnc959, lnc171, and lnc85), comparing their expression with HCC cell lines and exosome-derived from cell lines. From these lncRNAs, the inhibition of lnc85 resulted in the impairment of cell proliferation, and it decreased the migration in HCC cells. Functional analysis showed that lnc85 promoted proliferation, metastasis, and apoptosis of HCC cells by regulating the expression of miR-324-5p-associated target genes, thus showing this lncRNA as the sponge of miR-324-5p [116].

Another 2020 microarray study was reported by Lu et al. comparing exosomes derived from four groups of healthy control, chronic hepatitis, HCC without metastasis, and HCC with metastasis. Based on a Venn diagram of shared lncRNAs, they proposed six lncRNA candidates as biomarker predictors of tumorigenesis and metastasis of HCC, the ENSG00000248932.1, ENST00000440688.1, and ENST00000457302.2 LOC_001120, ENSG00000243766.2, AC058791.2, and TCONS_00003661. In a larger data set, three of the first lncRNAs were then confirmed in another 180 HCC cases as a validation set, confirmed with a continuously increasing level in healthy control, chronic hepatitis, and HCC groups. Interestingly, these lncRNAs also showed diagnostic values, with the AUC of 0.794, 0.571, and 0.538 for ENSG00000248932.1, ENST00000440688.1, and ENST00000457302.2, respectively. The combinations of these three lncRNAs showed an AUC of 0.838 [117].

Besides novel lncRNAs discovery by global screening, comparison studies, also supported by literature review, have demonstrated the presence of other lncRNAs in exosomes as a diagnostic tool. As a promising lncRNA, HULC has been demonstrated to be a strong diagnostic biomarker for HCC in various studies, as mentioned above. The presence of HULC in circulation can be derived also from exosomes. HULC expression in serum exosomes was related to its expression in HCC tissues, being higher in HCC (*n* = 30) compared to healthy controls. Both serum exosome and tissue level were correlated with the tumor-node-metastasis (TNM) classification. In HCC cell lines HepG2 and SMMC7721, the upregulation of HULC promoted HCC cell growth and invasion and repressed apoptosis, and at the same time decreased (sponged) the level of miR-372-3p [118].

In 2018, a study from Sun et al. showed LINC00161, together with LINC00462 and UCA1, being lncRNA upregulated in HCC serum samples (56 HCC vs. 56 controls; AUC = 0.794). Further examination on the exosome-enriched fraction (30–150 nm) showed that the levels of LINC00161 were upregulated in patients with HCC in contrast to controls (*p* < 0.05). No statistical significance was detected for the expression of LINC00161 in HCC urine and serum exosome-free samples compared with controls [99]. Previously, LINC00161 was shown to be expressed significantly higher in HCC tissues and its high expression was related to a poor prognosis [119].

The selection of lncRNA-RP11-583F2.2 targeting hsa-miR-1298 was based on bioinformatics tools named TCNG and NRED, with a high number of target genes. Exosomal lncRNAs were extracted from sera samples of 60 HCC, 42 chronic HCV, and 18 healthy donor individuals. Compared with the non-malignant groups, the malignant group (HCC) had a higher expression of lncRNA-RP11-583F2.2, with diagnostic power to distinguish HCC with healthy donors with AUC = 0.946 and sens/spec 97/92%. The use of this lncRNA was shown to reduce false-negative results, as compared to AFP alone [120].

Recently, the same group showed another related lncRNA, the lnc-RNA RP11-156p1.3 being significantly altered in HCC patients compared to chronic HCV patients and healthy controls. Functional analysis of this lncRNA was demonstrated by the use of CRISPR cas9 knock-out in HepG2 cells, which showed that the level of the genetic network was reversed and the protein levels of TNF α and NFκβ were decreased, together with a significant decrease in cell count and viability. It indicated that lnc-RNA RP11-156p1.3 can be a promising therapeutic target as well as a diagnostic tool [121].

The effect of HCV in exosomes was presented by lncRNA-HEIH. By using groups of chronic hepatitis C, HCV-related cirrhosis, and HCV-related HCC, the lncRNA-HEIH expression was found increased in the serum and exosomes of HCC groups. Interestingly, the ratio of lncRNA-HEIH expression in serum versus exosomes was decreased compared to patients with chronic hepatitis [122]. In in vitro model, HEIH is involved in HCC cell growth and metastasis through the regulation of several miRNAs, such as miR-199a-3p and miR-98-5p, and the deactivation of AKT and mTOR pathways [123,124,125].

### 3.2. Exosome-Derived CircRNAs

Through microarray profiling of plasma exosomal circRNAs, comparing three HCC patients with three healthy controls, Chen and colleagues identify a signature of 36 circRNAs able to cluster HCC and controls in two separated groups [64]. Among the 28 downregulated circRNAs in HCC, circ-0051443 was further confirmed as a biomarker candidate able to distinguish 60 HCC patients from controls (AUC = 0.809). Authors hypothesized a protective role for this circRNA, since its exosomal levels in exosomes of both healthy subjects and in normal cells circ-0051443 levels were high. Interestingly, its expression levels were four times higher in exosomes compared to producer cells [64]. Further investigations evidenced how circ-0051443 protects the cells from malignant transformation by sponging miR-331-3p [107] responsible for the targeting of B cell lymphoma (Bcl)-2 homologous antagonist/killer (Bak1), an essential cell death regulator, which initiates mitochondria-mediated apoptosis [126].

Despite the work from Li [99] being focused on the identification of the molecular effects derived from the transfer of exosomal circ-ZNF652 in cancer recipient cells, where the miR-29a-3p sponging promoted proliferation, migration, invasion, and glycolysis by affecting the guanylyl cyclase domain containing 1 (GUCD1) expression, it provided also evidence of circ-ZNF652 being higher in exosomes collected from cancer patients, compared to controls [99]. However, this evidence was derived from the analysis of 33 HCC samples and no further investigation of its diagnostic or prognostic potential was conducted.

Likewise, the expression of circ-FBLIM1 was investigated in 30 HCC samples compared to 25 healthy volunteers by Lai and colleagues [100]. The observation of high circ-FBLIM1 levels in HCC patients led the authors to hypothesize a relevant role for this circRNA in tumors. Their findings verified that circFBLIM1 contributed to the progression and glycolysis of HCC by sponging miR-338 and determining the consequent upregulating of LDL Receptor Related Protein 6 (LRP6) [100]. Despite the evidence of the high levels of circ-FBLIM1 in HCC exosomes, its value as a tumor biomarker should be further validated.

The circulating exosomal circAKT3, derived from the back splicing of exon 8–11 of the AKT3 gene, was significantly higher in HCC patients compared to healthy subjects. in patients with HCC, circAKT3 was associated with tumor sizes, microinvasion, and higher AFP levels, suggesting its possible role in this disease [104]. Besides the differential expression between healthy subjects and HCC patients, exosomal circAKT3 showed also some prognostic potential. Higher levels were associated with shorter survival time and shorter recurrence-free survival rate after surgery. Indeed, patients with high levels of exosomal circAKT3 showed higher recurrence (HR, 3.14; 95%CI 1.29–6.21, *p* = 0.012) and higher mortality (HR, 1.89; 95% CI, 1.04–3.01) [104]. This evidence suggested not only a possible role of this circRNA as a diagnostic biomarker for HCC derived from different etiologies, but also for the follow-up of patients receiving surgery to early identify recurrence episodes.

Intriguing is the role of exosomal circPTGR1 in HCC. In their study, Wang and colleagues identified three circRNAs, hsa_circ_0008043, hsa_circ_0003731, and hsa_circ_0088030, collectively named circPTGR1, since they are all transcribed from the same gene (prostaglandin reductase 1, PTGR1). They were significantly enriched in exosomes released by high metastatic LM3 cells, compared to HepG2 (a non-metastatic cell line) and 97L cells (a low-metastatic cell line) [105]. Interestingly, the incubation of HepG2 and 97L cells with LM3-derived exosomes enhanced the cell migration and invasion abilities of both cell lines, wowever, without affecting their proliferation, apoptosis, or cell cycle. When investigated in serum exosomes collected from HCC and healthy subjects, hsa_circ_0008043 and hsa_circ_0088030, but not hsa_circ_0003731, showed an up-regulation in HCC patients, compared to controls [105]. In addition, circPTGR1 differed significantly between clinical stages of HCC, being higher at TNM stage III. In agreement with these findings, the high levels of circPTGR1 was also associated with poorer survival. In vitro investigations identified the role of circPTGR1 in promoting MET proto-oncogene receptor tyrosine kinase (MET) expression by competing with the binding to miR449a, thus facilitating cellular survival, migration, and invasion [105].

Similar to the aforementioned example, circUHRF1 showed a clear association with the presence of a tumor [106]. It was up-regulated in exosomes isolated from 240 HCC patients compared to 20 healthy individuals. Moreover, its expression decreased with the surgical removal of the tumoral portion, and re-increased with tumor recurrence phenomena, thus suggesting circUHRF1 as a hallmark of liver cancer. In their study, Zhang and colleagues unrevealed one of the possible functions of the circulating exosomal circUHRF1. They found NK cells able to internalize HCC-derived exosomes loaded/enriched in circUHRF1. NK cells have been verified to have cytotoxic effects against tumor cells in several cancers [127,128], this activity was largely influenced by the expression of the inhibitory molecule programmed cell death 1(PD1). The T cell immunoglobulin and mucin domain 3 (TIM-3) is an important coinhibitory molecule is also expressed on NK cell and its expression is higher in NK cells from tumor patients than from healthy donors [129]. In this scenario, exosomal circUHRF1 internalized by NK cells could sustain the TIM-3 expression by sponging its inhibitor miR-449-5p [106], resulting in a suppression of the production of interferon γ (IFN-γ) and tumor necrosis factor α (TNF-α) by the cytotoxic NK cells [106]. These observations provide evidence of circUHRF1 as cancer-related circRNA able to identify patients that are likely to better respond to anti-PD1 treatments.

In exosomes collected from 30 HCC patients, the circTMEM45A (hsa_circ_0066659) was approximately four times higher compared to the exosomes collected from 30 healthy individuals. Despite no clinical significance was given to the high exosomal circTMEM45A levels, in vitro experiments proved the oncogenic role of this circRNA in HCC cells. Indeed, it had a role in sponging miR-665, thus determining tumor growth due to the up-regulation of the miR target insulin-like growth factor 2 (IGF2) [107].

## 4. Circulating Cells-Derived lncRNAs and CircRNAs

The peripheral blood mononuclear cells (PBMCs) include a variety of leukocyte subpopulations, such as monocytes, natural killer cells, T lymphocytes, B lymphocytes, and dendritic cells. Since the well-established role of immune cells in cancers, including HCC [130], many studies proposed PBMCs as a source of information related to the presence of the tumor, including prognosis and treatment response [131,132,133] for other molecules, also the altered expression profile of ncRNAs in PBMCs might provide some promising biomarkers for tumor diagnosis and management.

XIST and TSIX are two lncRNAs with a pivotal role in X chromosome inactivation (XCI). XIST (X inactive-specific transcript) was firstly reported in 1991, expressed specifically from inactive X chromosomes [134]. In 1999, TISX was discovered as gene antisense to XIST. It is a 40 kb RNA originating 15 kb downstream of XIST and transcribed across the XIST locus [135]. In HCC, XIST was involved in the cancer progression by sponging various miRNAs (e.g., miR-200b-3p, miR-155-5p, and miR-194-5p) [136,137]. In 2017, Ma et al. showed an increase of XIST expression in PBMC and granulocytes in HCC female patients. XIST had a significant discriminatory power to differentiate early-stage HCC group with healthy, CHB, and cirrhosis, compared to AFP [51]. They also showed that Jpx, another lncRNA, could be delivered from HCC cells to blood cells via exosomes and activate Xist expression of blood cells [51].

Hox antisense intergenic RNA (HOTAIR), is an oncogenic molecule localized to the HOXC gene cluster. It is one of the HCC markers since patients with high HOTAIR expression had significantly poorer prognoses and larger primary tumor size [138,139]. A study from Fujisaka [140] showed that CCL2, a monocyte chemoattractant protein 1, was differentially expressed in the HOTAIR-expressing liver cells. A co-culture of these cells with PBMC increased the proportion of CD14+HLA-DR+CD68+ macrophages [140].

Lei and colleagues [58] identified 58 circRNAs significantly dysregulated in the PBMCs collected from HCC patients compared with those of the healthy individuals. Six of them were further validated in 72 PBMCs samples from HCC patients and 30 from control subjects confirming the up-regulation of hsa-circ_0005505, hsa_circ_0000798, and hsa_circ_0001394 and the downregulation of hsa_circ_0001074, hsa_circ_0004771, and hsa_circ_0067735 in HCC-derived PBMC, compared to healthy controls. The increase of hsa_circ_0000798 in PBMC was also associated with a shorter survival rate. Patients with high has_circ_0000798 had a median survival of 10 months compared to the 24 months of patients having low levels has_circ_0000798. Additionally, some diagnostic potential was observed for circ_0000798 that discriminated HCC patients from healthy individuals with an AUC of 0.703 [58].

## 5. Future Message

Liquid biopsy for early HCC detection is a potent non-invasive strategy that leads to better clinical management of the disease. Scientific evidence in recent years indicated that lncRNAs and circRNAs can be good biomarkers due to their reliability and easy detection by PCR-based technology.

Some considerations are needed to use their potentials as HCC biomarkers. First is the standardization among laboratories. One of the problems of the RNA expression is due to technical variations among laboratories, including the reference genes used, the protocol, and the determination of cut-off value. The second problem is whether the circulating RNAs could specifically represent liver pathology, such as AFP which is a characterized liver protein. Several lncRNAs reviewed in this article are common in cancers, and not specific to HCC only. The third is whether these RNAs can be used to detect early stages of HCC (BCLC 0 and A), also with a single small nodule. Early diagnosis would allow the physician to apply curative treatments increasing the good prognosis of the patients. And last but not least, is the determination of which RNAs are the best marker, whether a single RNA, multiple RNA panels, and scoring values can be the strongest diagnostic determinant of HCC. Until the writing of this article, there is still substantial growth in the public database on the function and possible use of dozens lncRNAs and circRNAs, both in the tissues and in the circulation as a cancer diagnosis biomarker.

In summary, biomedical technology is growing fast and the discovery of cancer biomarkers is abundant. It is important to cross-check and validate these markers with various cohorts of patients representing high variabilities of HCCs, including patients with different etiologies, HCC cellular profiles, and stages of cancer. It should be noted whether the current therapy regimen applied to patients could alter the expression of these RNAs, thus providing their potential as a biomarker for patient follow-up and surveillance.

## Figures and Tables

**Table 2:** Circular RNAs (circRNAs) expression in HCC.

circRNA	Host Gene	Exp	Sponged miRNA	Source	n Samples ^a^	Ref
hsa_circ_0005505	Interleukin 1 Receptor Associated Kinase 3 (IRAK3)	up		PBMC	30/-/72	[58]
hsa_circ_0000798	Bromodomain PHD Finger Transcription Factor (BPTF)	up		PBMC	30/-/72	[58]
hsa_circ_0001394	TBC1 Domain Family Member 14 (TBC1D14)	up		PBMC	30/-/72	[58]
hsa_circ_0001074	Origin Recognition Complex Subunit 4 (ORC4)	down		PBMC	30/-/72	[58]
hsa_circ_0004771	Nuclear Receptor Interacting Protein 1 (NRIP1)	down		PBMC	30/-/72	[58]
hsa_circ_0067735	Mediator Complex Subunit 12L (MED12L)	down		PBMC	30/-/72	[58]
hsa_circ_104075	Nucleoporin 153 (NUP153)	up	miR-582-3p	serum	60/-/101	[59]
hsa_circ_0008234; circFOXP1	Forkhead Box P1 (FOXP1)	up	miR-875-3p and miR-421	serum	16/-/30	[60]
hsa_circ_0003258; circZNF652	Zinc Finger Protein 652 (ZNF652)	up	miR-29a-3p	exosomes	33/-/33	[99]
hsa_circ_0010090; circFBLIM1	Filamin Binding LIM Protein 1 (FBLIM1)	up	miR-338	exosomes	33/-/33	[100]
hsa_circ_101237	Cyclin-Dependent Kinase (CDK)8	up		serum	124/-/234	[101]
CircSMARCA5	SWI/SNF Related, Matrix Associated, Actin Dependent Regulator of Chromatin, Subfamily A, Member 5 (SMARCA5)	down		plasma	103/117+143/135	[61]
circZEB1.33	Zinc Finger E-Box Binding Homeobox 1(ZEB1)	up	miR-200a-3p	serum	30/-/64	[102]
hsa_circ_0003998	ADP Ribosylation Factor Guanine Nucleo- Tide Exchange Factor 2 (ARFGEF2)	up		plasma	50/50/100	[62]
hsa_circ_0027089	Prostaglandin E Synthase 3 (PTGES3)	up		plasma	72/64/84	[63]
hsa_circ_0077930	Abelson Helper Integration Site 1 (AHI1)	up		plasma	-/24/24	[63]
hsa_circ_0001818	Ubiquitin Protein Ligase E3 Component N-Recognin 5 (UBR5)	up		plasma	-/24/24	[63]
hsa_circ_0026337	Sodium Voltage-Gated Channel Alpha Subunit 8 (SCN8A)	up		plasma	-/24/24	[63]
hsa_circ_0011883	Palmitoyl-Protein Thioesterase 1 (PPT1)	down		plasma	-/24/24	[63]
hsa_circ_0001070	R3H Domain Containing 1 (R3HDM1)	down		plasma	-/24/24	[63]
hsa_circ_0064428	Solute Carrier Family 6 Member 6 (SLC6A6)	down		plasma	56/64 (high vs. low TILs)	[103]
hsa_circ_0055538	Required for Meiotic Nuclear Division 5 Homolog A (RMND5A)	down		plasma	56/64 (high vs. low TILs)	[103]
hsa_circ_0065964	ABHD14A-ACY1 Readthrough	up		plasma	56/64 (high vs. low TILs)	[103]
hsa_circ_0011386	Eukaryotic Translation Initiation Factor 3 Subunit I (EIF3I)	up		plasma	56/64 (high vs. low TILs)	[103]
hsa_circ_0044172	Mitogen-Activated Protein Kinase Kinase Kinase 14 (MAP3K14)	up		plasma	56/64 (high vs. low TILs)	[103]
hsa_circ_0010882	Ribosomal Protein L1 (RPL1)	up		plasma	56/64 (high vs. low TILs)	[103]
hsa_circ_0000199, circAKT3	AKT Serine/Threonine Kinase 3 (AKT3)	up		exosomes	100/-/124	[104]
hsa_circ_0088030, circPTGR1	Prostaglandin Reductase 1 (PTGR1)	up	miR-449a	exosomes	47/-/82	[105]
hsa_circ_0008043, circPTGR1	Prostaglandin Reductase 1 (PTGR1)	up	miR-449a	exosomes	47/-/82	[105]
circUHRF1	Ubiquitin Like with PHD and Ring Finger Domains 1 (UHRF1)	up	miR-449-5p	exosomes	20/-/240	[106]
hsa_circ_0051443	Homo Sapiens Trafficking Protein Particle Complex 6A (TRAPPC6A)	down	miR-331-3p	exosomes	60/-/60	[64]
hsa_circ_0066659 circTMEM45A	Transmembrane Protein 45A	up	miR-665	exosomes	30/-/30	[107]
hsa_circ_0004001	CDC Like Kinase 1 (CLK1)	up		serum	40/-/71	[65]
hsa_circ_0004123	ETS Variant Transcription Factor 6 (ETV6)	up		serum	40/-/71	[65]
hsa_circ_0075792	Lysine Demethylase 1B (KDM1B)	up		serum	40/-/71	[65]
hsa_circ_0009582	Arginine-Glutamic Acid Dipeptide Repeats (RERE)	up		plasma	200/200/200	[66]
hsa_circ_0037120	Rhomboid 5 Homolog 1 (RHBDF1)	up		plasma	200/200/200	[66]
hsa_circ_0140117	Connector Enhancer of Kinase Suppressor of Ras 2 (CNKSR2)	up		plasma	200/200/200	[66]
hsa_circ_0000976	Hippocalcin Like 1 (HPCAL1)	up		plasma	179/186+180/600	[108]
hsa_circ_0007750	Rab Geranylgeranyltransferase Subunit Alpha (RABGGTA)	up		plasma	179/186+180/600	[108]
hsa_circ_0139897	Myotubularin 1 (MTM1)	up		plasma	179/186+180/600	[108]

^a^ Sample (HCC/chronic&cirrhosis/healthy), TILs = tumor-infiltrating lymphocytes.

**Table 3:** List of small non-coding RNA (sncRNA) panel for HCC diagnosis and in combination with alpha-fetoprotein (AFP).

sncRNA Panel	AUC ^a^	sncRNA Panel + AFP	AUC ^a^	Ref
**lncRNA**				
-	-	LINC00152, PTENP1, UCA1, and AFP	0.912	[41]
LINC00152, RP11-160H22.5, XLOC014172	0.985	LINC00152, RP11-160H22.5, XLOC014172, AFP	0.986	[42]
HULC, LINC00152	0.870	HULC, LINC00152, AFP	0.890	[39]
SNGH1	0.920	SNHG1, AFP	0.970	[54]
PVT1, uc002mbe.2	0.764	PVT1, uc002mbe.2, AFP (20ng/mL)	0.831	[110]
RP11–160H22.5, XLOC_014172, LOC149086	0.896	-	-	[45]
uc001ncr, AX800134	0.949	-	-	[57]
SPRY4-IT1	0.702	SPRY4-IT1, AFP	0.800	[28]
UCA1, WRAP53		UCA1, WRAP53, AFP		[49]
JPX		JPX, AFP	0.905	[50]
				
**circRNA**				
hsa_circ_0004001+hsa_circ_0004123+ hsa_circ_0075792	0.89			[65]
hsa_circ_0009582+ hsa_circ_0037120hsa_circ_0140117	Sen/spe 80/95 vs. CH 84/80 vs. cirr			[66]
hsa_circ_0000976+hsa_circ_0007750+hsa_circ_0139897	0.860			[108]
hsa_circ_0027089	0.790	hsa_circ_0027089, AFP	0.800	[63]

^a^ vs healthy control.

## Data Availability

Data sharing not applicable. No new data were created or analyzed in this study. Data sharing is not applicable to this article.
